# Hepatitis B (HBV), Hepatitis C (HCV) and Hepatitis Delta (HDV) Viruses in the Colombian Population—How Is the Epidemiological Situation?

**DOI:** 10.1371/journal.pone.0018888

**Published:** 2011-04-29

**Authors:** Mónica Viviana Alvarado-Mora, María Fernanda Gutierrez Fernandez, Michele Soares Gomes-Gouvêa, Raymundo Soares de Azevedo Neto, Flair José Carrilho, João Renato Rebello Pinho

**Affiliations:** 1 Laboratory of Gastroenterology and Hepatology, São Paulo Institute of Tropical Medicine and Department of Gastroenterology, School of Medicine, University of São Paulo, São Paulo, Brazil; 2 Laboratory of Virology, Department of Microbiology, Pontificia Javeriana University, Bogotá, Colombia; 3 Department of Pathology, School of Medicine, University of São Paulo, São Paulo, Brazil; Copenhagen University Hospital Gentofte, Denmark

## Abstract

**Background:**

Viral hepatitis B, C and delta still remain a serious problem worldwide. In Colombia, data from 1980s described that HBV and HDV infection are important causes of hepatitis, but little is known about HCV infection. The aim of this study was to determine the currently frequency of HBV, HCV and HDV in four different Colombian regions.

**Methodology/Principal Findings:**

This study was conducted in 697 habitants from 4 Colombian departments: Amazonas, Chocó, Magdalena and San Andres Islands. Epidemiological data were obtained from an interview applied to each individual aiming to evaluate risk factors related to HBV, HCV or HDV infections. All samples were tested for HBsAg, anti-HBc, anti-HBs and anti-HCV markers. Samples that were positive to HBsAg and/or anti-HBc were tested to anti-HDV. Concerning the geographical origin of the samples, the three HBV markers showed a statistically significant difference: HBsAg (p = 0.033) and anti-HBc (p<0.001) were more frequent in Amazonas and Magdalena departments. Isolated anti-HBs (a marker of previous vaccination) frequencies were: Chocó (53.26%), Amazonas (32.88%), Magdalena (17.0%) and San Andrés (15.33%) - p<0.001. Prevalence of anti-HBc increased with age; HBsAg varied from 1.97 to 8.39% (p = 0.033). Amazonas department showed the highest frequency for anti-HCV marker (5.68%), while the lowest frequency was found in San Andrés Island (0.66%). Anti-HDV was found in 9 (5.20%) out of 173 anti-HBc and/or HBsAg positive samples, 8 of them from the Amazonas region and 1 from them Magdalena department.

**Conclusions/Significance:**

In conclusion, HBV, HCV and HDV infections are detected throughout Colombia in frequency levels that would place some areas as hyperendemic for HBV, especially those found in Amazonas and Magdalena departments. Novel strategies to increase HBV immunization in the rural population and to strengthen HCV surveillance are reinforced by these results.

## Introduction

Around two billion people worldwide have been infected by hepatitis B virus (HBV) and approximately 350 million people are chronic carriers. Every year about one million deaths are caused by chronic HBV infection [1]. Approximately 400.000 new cases are estimated to occur in Latin America each year and 10 to 70% of them may evolve to hepatocellular carcinoma [2]. Considering the HBV infection epidemiology throughout different geographical zones, the world is divided in high, intermediate and low endemicity areas, corresponding to infection rates higher than 8%, 2 to 8% and lower than 2%, respectively [3].

The routes of HBV transmission in South and Central America are highly variable. The highest prevalence was reported in 20 to 40 years old age group, and adult horizontal transmission is the most common route of infection [4]. Furthermore, vertical and childhood horizontal transmission are also important in areas of high endemicity such as the Amazon Basin [5], [6].

HBV strains have distinct geographical distribution that are classified in nine genotypes (A to I) based on genome diversity and some of them are further classified in subgenotypes [7]. In Colombia, subgenotype F3 is the most common subgenotype among the HBV infected patients, but it was also reported an important frequency of genotypes E, A2, G and F1b among HBV chronic patients [8], [9].

Hepatitis D virus (HDV) is a subviral agent that requires a preexisting or concurrent infection with HBV [10]. HDV genome is a 1672 to 1697 nucleotides circular single-strand RNA of with extensive intramolecular complementarity [11], [12]. Compared with infection with HBV alone, HDV coinfection with HBV is associated with a higher rate of fulminant hepatitis in an acute infection and HDV superinfection in individuals with chronic HBV infection can lead to more severe progressive chronic liver disease [10].

HDV is classified in eight different genotypes: genotype 1 is the most frequent and found in Europe, Middle East, North America and North Africa [13]–[15]; genotype 2 is seen in the Far East; genotype 3 was reported in the Amazonian region of South America [16], [17]; genotype 4 was isolated in Taiwan and Japan [18]–[20]; and genotypes 5 to 8 have been identified in Africans [21]. HDV is distributed worldwide with more than 24% of HBV carriers with HDV markers present in Africa, Southwest Asia and in the Mediterranean basin. In the United States, HDV prevalence is lower, ranging from 1 to 10% [22]. In Colombia, few reports are available on HDV prevalence, mainly showing its association to fulminant hepatitis outbreaks in departments with intermediate or high HBV endemicity, particularly in the Amazonas department where high HDV antibodies prevalence rates have been found among children younger than 4 years old [23].

Hepatitis C virus (HCV) infection is a public health problem throughout the world infecting around 3% of the world population. HCV is classified in six major genotypes and more than 70 subtypes [24], [25]. It is an enveloped, single stranded positive sense RNA virus with a 50 nm diameter viral particle, classified in *Hepacivirus* genus of the *Flaviviridae* family [26]. Countries with the highest reported prevalence rates are located in Africa and Asia; while lower prevalence areas include North America, North and West Europe, and Australia [27]. In Latin America, HCV overall prevalence is 1.23% [4]. However, the HCV antibody in blood donors in Latin America varies from 0.2% –0.5% in Chile to 1.7% –3.4% in northeast of Brazil [4], [28], [29]. The main risk factors are unsafe injection techniques and blood transfusions [29]. Mandatory screening for HCV infection was gradually adopted in Latin America during the 1990s, leading to a currently decreased blood transmission in most South American countries [4], [29]. One study carried out in Colombia during 2005 showed a prevalence of 9% of HCV among multi-transfused patients [30]. The distribution of HCV genotypes in Colombia showed that subtype 1b is the most prevalent and that HCV emerged in Bogotá around 60 years ago [31].

Since in Colombia there are few epidemiologic studies about the hepatitis virus, the aim of this study was to determine the frequency of serological markers for viral hepatitis B, C and D (delta) in four regions in Colombia and to analyze the risk factors associated with viral transmission in these populations.

## Methods

### Study population

**Figure 1:**
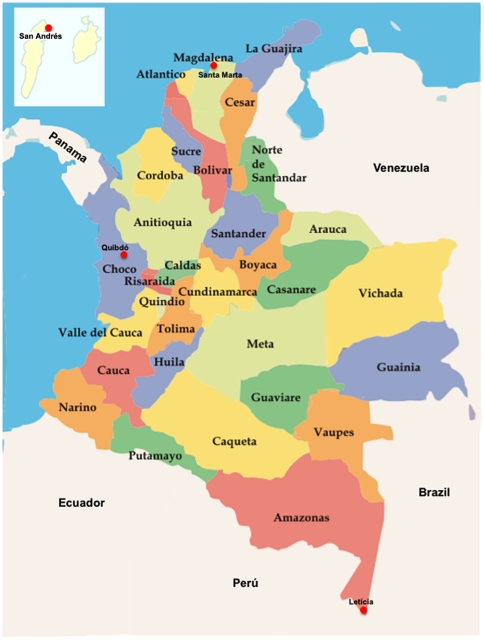
Geographic localization of the four places from where samples were collected in Colombia. The capital of each department is in red (•).

This study was conducted in 697 habitants with ages varying from 12 to 72 years old from the rural and urban areas of four departments in Colombia: Amazonas (n = 184), Chocó (n = 138), Magdalena (n = 218) and San Andres Islands (n = 156) (Figure 1). Epidemiological data were obtained from an interview applied to each individual aiming to evaluate risk factors related to HBV, HCV or HDV infections. The Ethical Committees of the Pontificia Universidad Javeriana, Bogotá, Colombia and the University of São Paulo Medical School, São Paulo, Brazil, approved this protocol. All patients have signed an informed consent form before joining the study.

Sampling was carried out for each department during the span of a week. The individuals were summoned in the public health laboratory from each region. Different risk groups were evaluated in this study. In the Amazonas department, sampling was performed in the area of the Amazon River, involving Amerindian populations originating from four different ethnic groups (Ticuna, Huitoto, Huinane, and Yagua). In Chocó department and San Andres Islands, sampling included female sex workers, doctors and nurses. In Magdalena department, it included medical workers and “desplazados” populations (i.e., people that were obliged to leave their homes due to the civil war).

### Serological tests

All samples were tested for HBsAg, anti-HBc, anti-HBs and anti-HCV markers. Samples that were positive to for HBsAg and/or anti-HBc were tested to anti-HDV. These assays were performed using commercial available ELISA kits (DiaSorin, Italy).

### Statistical analyses

Statistical analyses were performed using Minitab Software v. 15. The χ^2^ test for linear trend (α = 0.05) was used to examine the distribution of HBV, HCV and HDV markers according to age group, sex, and geographical origin. Results were considered statistically significant when p<0.05.

## Results

The results for all serological markers tested in the samples and for the statistical analysis according to three analyzed variables (sex, age group and geographical origin of the samples) are shown in Table 1.

**Table 1:** Results for each HBV, HDV and HCV serology marker evaluated in the Colombian population (>11 years old).

Variables	HBsAg Positives/n (%)	anti-HBc Positives/n (%)	anti-HBs Positives/n (%)	Isolated anti-HBs Positives/n (%)	anti-HDV Positives n (%)	anti-HCV Positives/n (%)
***Sex***						
Male group	7/163 (4.29%)	56/163 (34.36%)	73/149 (48.99%)	33/96 (34.38%)	3/56 (5.36%)	6/163 (3.68%)
Female group	28/455 (6.15%)	120/456 (26.32%)	175/427 (40.98%)	78/306 (25.49%)	6/117 (5.13%)	16/456 (3.51%)
*p*	0.378	0.051	0.089	0.089	0.949	0.919
***Age group (years)***						
11–15	1/32(3.13%)	0/32(0.00%)	6/30 (20.00%)	6/30 (20.00%)	no samples	0/32(0.00%)
16–20	5/58(8.62%)	7/58 (12.07%)	19/54 (35.19%)	11/45 (24.44%)	0/7 (0.00%)	1/58 (1.72%)
21–25	8/130(6.15%)	25/130 (19.23%)	43/116 (37.07%)	25/94 (26.60%)	1/22 (4.55%)	6/130 (4.62%)
26–30	8/97(8.25%)	26/97 (26.80%)	44/94 (46.81%)	23/66 (34.85%)	0/23 (0.00%)	7/97 (7.22%)
31–35	4/69(5.80%)	20/69 (28.99%)	29/64 (45.31%)	12/45 (26.67%)	1/22 (4.55%)	2/69 (2.90%)
36–40	0/61(0.00%)	20/62 (32.26%)	26/58 (44.83%)	11/38 (28.95%)	1/20 (5.00%)	0/62(0.00%)
41–45	2/55(3.64%)	19/55 (34.55%)	21/52 (40.38%)	7/33 (21.21%)	3/20 (15.0%)	2/55 (3.64%)
46–50	3/35(8.57%)	17/35 (48.57%)	19/33 (57.58%)	5/16 (31.25%)	1/18 (5.56%)	0/35(0.00%)
>51	4/81(4.94%)	42/81 (51.85%)	41/75 (54.67%)	11/35 (31.43)	2/41 (4.88%)	4/81 (4.94%)
*p*	0.502	<0.001	0.026	0.860	*	0.259
***Geographical Origin***						
Amazonas	14/176 (7.95%)	96/176 (54.55%)	105/168 (62.50%)	24/73 (32.88%)	8/97 (8.25%)	10/176 (5.68%)
Chocó	5/135 (3.70%)	25/136 (18.38%)	67/115 (58.26%)	49/92 (53.26%)	0/27 (0.00%)	5/136 (3.68%)
San Andrés Island	3/152 (1.97%)	6/152 (3.95%)	25/143 (17.48%)	21/137 (15.33%)	0/9 (0.00%)	1/152 (0.66%)
Magdalena	13/155 (8.39%)	49/155 (31.61%)	51/150 (34.00%)	17/100 (17.00%)	1/40 (2.50%)	6/155 (3.87%)
*p*	0.033	<0.001	<0.001	<0.001	*	0.107
**Total**	**35/618 (5.66%)**	**176/619 (28.43%)**	**248/576 (43.06%)**	**111/402 (27.61%)**	**9/173 (5.20%)**	**22/619 (3.55%)**

*p value was not calculated due to the small number of positive samples.

### Hepatitis B virus markers (HBsAg, anti-HBc and anti-HBs)

In spite of the higher frequency of anti-HBc and anti-HBs positive cases when comparing males to females patients, these differences were not statistically significant (p = 0.051 and p = 0.089, respectively). Anti-HBc (p<0.001) frequency increased with age, from the 11–15 years old group to the >51 years old group. Anti-HBs (p = 0.026) frequency was also different in the several age groups and was higher in the 46–50 and >51 years old groups. HBsAg frequency was not different according to sex and age (p = 0.378 and p = 0.502, respectively).

Amazonas and Magdalena departments showed the highest frequency for all HBV markers while San Andres Islands and Chocó showed a low frequency for them. The differences for HBsAg (p = 0.033), anti-HBc (p<0.001) and anti-HBs (p<0.001) frequencies in the various geographical origins of the samples were statistically significant.

Concerning the geographical origin of the samples, the three HBV markers showed a statistically significant difference. HBsAg (p = 0.033) and anti-HBc (p<0.001) were more frequent in Amazonas and Magdalena departments and less frequent in San Andrés Islands and Chocó. For anti-HBs (p<0.001), the highest and lowest frequencies were also observed in Amazonas department and San Andrés Islands, respectively, but Chocó department showed the second highest frequency.

Another analysis was carried out to verify the frequency of isolated anti-HBs as a marker of previous vaccination. Its frequency by geographical origin of the samples was: Chocó (53.26%), Amazonas (32.88%), Magdalena (17.0%) and San Andrés (15.33%) - p<0.001. Isolated anti-HBs distribution was not different according to sex and age (p = 0.089 and p = 0.860, respectively).

### Hepatitis C marker (anti-HCV)

Amazonas department showed the highest frequency for anti-HCV marker (5.68%). The lowest frequency was found in San Andrés Island (0.66%) and intermediate levels were found in the two other departments, but these differences were not statistically significant (p = 0.107). Considering age groups, the higher frequency for anti-HCV was found from 26 to 30 (7.22%) years old, but it was not statistically significant (p = 0.259). Geographical origin of samples did not show statistically significant difference considering the presence of anti-HCV in the population (p = 0.107).

### Hepatitis Delta marker (anti-HDV)

One-hundred seventy three anti-HBc positive and/or HBsAg-positive samples were screened for anti-HDV. Nine (5.20%) samples (from 3 males and 6 females) were positive for this marker. Eight of these samples were from the Amazonas region and one sample came from Magdalena department. Anti-HDV positive patients ranged from 21 to 67 years old. No statistically significant differences were found for anti-HDV distribution and sex (p = 0.949). For age group and geographical origin of samples, it was not possible to carry out a statistical analysis due to the small number of positive samples.

## Discussion

In Colombia, there are a few studies about hepatitis epidemiology. This is the first currently study reporting HBV, HCV and HDV serological profiles in these four departments (Amazonas, Chocó, Magdalena and San Andrés Islands).

The overall anti-HBc and HBsAg frequencies found were 28.43% and 5.66%, respectively. These rates include Colombia as an intermediate endemicity region in at least these four regions that we have analyzed. HBsAg positivity rates ranged from 1.97% in San Andrés Islands to 8.39% in Magdalena department, slightly surpassing the intermediate endemicity rates (2 to 8%) in this last department.

Serological markers for HBV infection have been previously found to range from 25 to 83% among Amazonian peoples in Bolivia, Brazil, Peru and Venezuela [32], [33]. In a study performed between 1977 and 1989, overall HBsAg seroprevalence rate was 4.7% in Colombia [34].

In a previous paper analyzing the different Colombian regions, HBsAg prevalence was 7.1%, 3.5% and 2.8% in the Central region, in the Pacific zone and in the Eastern region, respectively [35]. In the present paper, we have analyzed Chocó, in the Pacific region, and we have found a HBV intermediate prevalence of 3.7% of the studied population, very close to the previously reported.

In another study carried out in 1975 involving native Chocó people who lived in the Darien forest, it was reported a higher exposure to HBV: 42% anti-HBs positive but only 1.2% HBsAg positive [36]. The prevalence of HBV markers among these native Chocó people is lower than that found in the Chocó population studied in the present paper as well as that found in other native people who live in the Amazon department, as shown below. This difference might be related to the higher sensitivity of the methodology applied nowadays.

Western Amazonas basin is reported as one region with the highest rates of hepatitis B infection in the world as more than 80% of people living in some rural areas have been infected with HBV and more than 8% carry HBsAg [4], [37], [38]. In the present paper, we are reporting 7.95% HBsAg positivity among the studied population from Amazonas department, confirming that HBV infection remains a problem in this region.

Near the capital of Magdalena department, Santa Marta city, in northern Colombia, recurrent epidemics of severe hepatitis have been reported over many years and have acquired the name of “Santa Marta hepatitis”. This hepatitis was described in 1930s [39] as a severe icteric illness with mortality as high as 10% [40]. HBsAg frequency found in Magdalena department in this study was 8.39%, the highest found among the 4 departments analyzed. Previous studies carried during the 80′s found HBsAg levels higher in studies of the populations affected with “Santa Marta Hepatitis” [34]. The present study could not study the population of Sierra Nevada de Santa Marta where these epidemics have been described but the finding of higher levels of HBV endemicity in this area shows that it is remains a public health problem in this area.

To our knowledge, previous data on the frequency of hepatitis viruses markers at San Andrés Islands have not been published before. These islands showed the lowest prevalence for all HBV and HCV markers among the four studied regions. HDV infection was not detected.

Anti-HBc positivity increased in older age groups. This finding agrees with the known role of sexual transmission in HBV spreading. As we did not have studied any children with less than 12 years old in this study, we cannot evaluate the role of vertical transmission for hepatitis infection in this population. Anti-HBs positivity also increased in older age groups, but surprisingly HBsAg positivity did not showed a statistically significant difference with age as some age groups showed particularly lower levels of HBsAg detection (especially the 36 to 40 years old age group).

Cuba, Colombia and Brazil were the first countries in Latin America that introduced universal HBV vaccination in the early 1990s [41]. Isolated anti-HBs (i.e., immunized after vaccination) individuals constitute only 17.96% (n = 111) of the studied population (n = 618). Considering only the susceptible individuals (n = 402), i.e., anti-HBc and HBsAg seronegatives but anti-HBs seropositives, only 27.61% of them were protected against HBV infection. Isolated anti-HBs was detected in more than half and in about one third of susceptible individuals in Chocó and Amazonas departments, respectively. In San Andrés Islands and Magdalena departments, isolated anti-HBs levels are only 15.33 and 17.00%, respectively. It is noteworthy that the highest HBsAg positive rate was detected in Magdalena department.

In 2002, it was reported a HBV vaccine coverage in Chocó about 26.2% [42]. In Chocó department, we found that 53.26% individuals with isolated anti-HBs, reflecting previous HBV vaccination, what would explain the HBsAg frequency found. At San Andrés Islands, we found a low frequency of isolated anti-HBs (15.3%) but also a lower frequency of HBV infection markers. Isolated anti-HBs frequency was 32.88% in Amazonas department.

These results show that HBV vaccination must be reinforced in Colombia as an effective health program for this population to prevent new hepatitis B cases. This is an important public health problem involving the costs for the implementation of widespread efficient vaccination programs in large regions inside scarcely inhabited areas where the populations are spread in small villages located in the middle of the equatorial rain forest. Furthermore, such program might face unexpected problems, as some also indigenous populations in the Amazonian region may not accept HBV vaccination for cultural reasons.

For hepatitis delta, areas of high prevalence include the Mediterranean Basin, the Middle East, Central Asia, Amazon Basin in South America and certain South Pacific islands [43]. Severe, often fatal, acute and chronic type D hepatitis occurs among indigenous people for Venezuela, Colombia, Brazil and Peru, all regions with high chronic HDV infection rates [44].

In Colombia, HDV infection is common in Amazonas and Magdalena departments [37], [38], [45], [46]. These data were confirmed in this study as the only anti-HDV positive samples found were from these two departments: 8 (8,25%)/97 and 1 (2.5%)/40 among HBsAg and/or anti-HBc positive samples from Amazonas and Magdalena departments, respectively. These two departments have a huge jungle region and HDV genotype 3 predominates [10], [17]. Buitrago et al., in 1986, suggested that HDV infection has been endemic in northern South America for more than 50 years. The high frequency of HBV, together with the presence of HDV markers, showed that these viruses represent the etiologic factors of the “hepatitis of the Sierra Nevada de Santa Marta” [40]. It is important to reinforce that a continuous surveillance of this area allied to an intense HBV vaccination program is needed to control this severe hepatic disease, as cases co-infected with both viruses are still being detected.

The estimates of HCV prevalence in Colombia correspond to data collected from blood donors, since there is no study of prevalence in the general population [30]. A study reporting the potential risk for an infectious disease through tainted transfusion involving Colombian blood donors reported that 98.30% of them were submitted to anti - HCV screening and 0.90% of those were anti - HCV positive in 1993. This study also reported in Colombia a probability of 74.55 per 10,000 donors of receiving an infected transfusion and 67.09 per 10,000 donors of getting a transfusion-transmitted infection, which was the highest risk of receiving a blood unit infected with HCV and of contracting this infection in Latin America [47]. Colombia has made screening for HCV mandatory since 1993 [48] and the coverage for serology for that infection in the blood banks has increased since now. In another study, the screening coverage for anti-HCV in Colombia in 2002 increased to 99.70% and the risks of receiving an infected unit or developing an infection after receiving an infected unit of blood in decreased to 0.24 per 10,000 donors and 0.22 per 10,000 donors, respectively [49]. These results strongly agree with previous results from our group showing that HCV genotype 1b, the most frequent in Colombia, exponentially spread up to 1992, when its growth was controlled by HCV screening in Blood Banks [31].

In this study, anti - HCV frequency ranged from 0.66% in San Andres Island up to 5.68% in Amazonas department. HCV is an infection that probably arrived recently in Colombia [31] when compared to HBV, which is found wide spread in Native Colombians people and some HBV genotypes are quite probably longer in South America [9]. Nevertheless, the widespread presence of this infection throughout the world, the known transmissions routes by blood supplies and other parenteral routes, led this infection to spread around the world and it is present in all the Colombians regions studied, in some of them in endemicity levels that deserve deeper attention of health policy authorities.

In conclusion, HBV, HCV and HDV infections are detected throughout Colombia in frequencies levels that would place some areas as hyperendemic for HBV, especially those found in Amazonas and Magdalena departments.

## References

[1] Lavanchy D (2004). Hepatitis B virus epidemiology, disease burden, treatment, and current and emerging prevention and control measures.. J Viral Hepat.

[2] Fay OH (1990). Hepatitis B in Latin America: epidemiological patterns and eradication strategy. The Latin American Regional Study Group.. Vaccine.

[3] Tanaka J (2000). Hepatitis B epidemiology in Latin America.. Vaccine.

[4] Te HS, Jensen DM (2010). Epidemiology of hepatitis B and C viruses: a global overview.. Clin Liver Dis.

[5] Torres JR (1996). Hepatitis B and hepatitis delta virus infection in South America.. Gut.

[6] Gish RG, Gadano AC (2006). Chronic hepatitis B: current epidemiology in the Americas and implications for management.. J Viral Hepat.

[7] Yu H, Yuan Q, Ge SX, Wang HY, Zhang YL (2010). Molecular and phylogenetic analyses suggest an additional hepatitis B virus genotype “I”.. PLoS One.

[8] Alvarado Mora MV, Romano CM, Gomes-Gouvea MS, Gutierrez MF, Carrilho FJ (2010). Molecular epidemiology and genetic diversity of hepatitis B virus genotype E in an isolated Afro-Colombian community.. J Gen Virol.

[9] Alvarado Mora MV, Romano CM, Gomes-Gouvea MS, Gutierrez MF, Botelho L (2011). Molecular characterization of the Hepatitis B virus genotypes in Colombia: a Bayesian inference on the genotype F.. Infect Genet Evol.

[10] Casey JL, Brown TL, Colan EJ, Wignall FS, Gerin JL (1993). A genotype of hepatitis D virus that occurs in northern South America.. Proc Natl Acad Sci U S A.

[11] Wang KS, Choo QL, Weiner AJ, Ou JH, Najarian RC (1986). Structure, sequence and expression of the hepatitis delta (delta) viral genome.. Nature.

[12] Radjef N, Gordien E, Ivaniushina V, Gault E, Anais P (2004). Molecular phylogenetic analyses indicate a wide and ancient radiation of African hepatitis delta virus, suggesting a deltavirus genus of at least seven major clades.. J Virol.

[13] Makino S, Chang MF, Shieh CK, Kamahora T, Vannier DM (1987). Molecular cloning and sequencing of a human hepatitis delta (delta) virus RNA.. Nature.

[14] Chao YC, Chang MF, Gust I, Lai MM (1990). Sequence conservation and divergence of hepatitis delta virus RNA.. Virology.

[15] Shakil AO, Hadziyannis S, Hoofnagle JH, Di Bisceglie AM, Gerin JL (1997). Geographic distribution and genetic variability of hepatitis delta virus genotype I.. Virology.

[16] Quintero A, Uzcategui N, Loureiro CL, Villegas L, Illarramendi X (2001). Hepatitis delta virus genotypes I and III circulate associated with hepatitis B virus genotype F In Venezuela.. J Med Virol.

[17] Gomes-Gouvea MS, Soares MC, Bensabath G, de Carvalho-Mello IM, Brito EM (2009). Hepatitis B virus and hepatitis delta virus genotypes in outbreaks of fulminant hepatitis (Labrea black fever) in the western Brazilian Amazon region.. J Gen Virol.

[18] Wu JC, Chiang TY, Sheen IJ (1998). Characterization and phylogenetic analysis of a novel hepatitis D virus strain discovered by restriction fragment length polymorphism analysis.. J Gen Virol.

[19] Sakugawa H, Nakasone H, Nakayoshi T, Kawakami Y, Miyazato S (1999). Hepatitis delta virus genotype IIb predominates in an endemic area, Okinawa, Japan.. J Med Virol.

[20] Watanabe H, Nagayama K, Enomoto N, Chinzei R, Yamashiro T (2003). Chronic hepatitis delta virus infection with genotype IIb variant is correlated with progressive liver disease.. J Gen Virol.

[21] Le Gal F, Gault E, Ripault MP, Serpaggi J, Trinchet JC (2006). Eighth major clade for hepatitis delta virus.. Emerg Infect Dis.

[22] Gaeta GB, Stroffolini T, Chiaramonte M, Ascione T, Stornaiuolo G (2000). Chronic hepatitis D: a vanishing disease? An Italian multicenter study.. Hepatology.

[23] Espinal C (1998). Perfil Epidemiológico de la Hepatitis B y D en Colombia.. Biomédica.

[24] Pasquier C, Njouom R, Ayouba A, Dubois M, Sartre MT (2005). Distribution and heterogeneity of hepatitis C genotypes in hepatitis patients in Cameroon.. J Med Virol.

[25] Echevarria JM, Leon P (2003). Epidemiology of viruses causing chronic hepatitis among populations from the Amazon Basin and related ecosystems.. Cad Saude Publica.

[26] Bostan N, Mahmood T (2010). An overview about hepatitis C: A devastating virus.. Critical Reviews in Microbiology.

[27] Shepard CW, Finelli L, Alter MJ (2005). Global epidemiology of hepatitis C virus infection.. Lancet Infect Dis.

[28] Carrilho FJ, Corrêa MCJM, Schinazi RF, Somadossi JP, Thomas HC (1998). Magnitude of hepatitis B and C in Latin America.. Therapies for viral hepatitis.

[29] Soza A, Riquelme A, Arrese M (2010). Routes of transmission of hepatitis C virus..

[30] Beltran M, Navas MC, De la Hoz F, Mercedes Munoz M, Jaramillo S (2005). Hepatitis C virus seroprevalence in multi-transfused patients in Colombia.. J Clin Virol.

[31] Mora MV, Romano CM, Gomes-Gouvea MS, Gutierrez MF, Carrilho FJ (2010). Molecular characterization, distribution, and dynamics of hepatitis C virus genotypes in blood donors in Colombia.. J Med Virol.

[32] Bensabath G, Hadler SC, Soares MC, Fields H, Maynard JE (1987). Epidemiologic and serologic studies of acute viral hepatitis in Brazil's Amazon Basin.. Bull Pan Am Health Organ.

[33] Hadler SC, De Monzon M, Ponzetto A, Anzola E, Rivero D (1984). Delta virus infection and severe hepatitis. An epidemic in the Yucpa Indians of Venezuela.. Ann Intern Med.

[34] Ljunggren KE, Patarroyo ME, Engle R, Purcell RH, Gerin JL (1985). Viral hepatitis in Colombia: a study of the “hepatitis of the Sierra Nevada de Santa Marta”.. Hepatology.

[35] Prieto F, Rojas D (2003). Situación semestral de la hepatitis B, Colombia. Programa ITS/sida, Instituto Nacional de Salud.. Biomédica.

[36] Reeves WC, Peters CJ, Purcell RH (1975). The epidemiology of hepatitis B antigen and antibody among Panamanian Cuna Indians.. Am J Trop Med Hyg.

[37] Gayotto LC (1991). Hepatitis delta in South America and especially in the Amazon region.. Prog Clin Biol Res.

[38] de la Hoz F, Martínez M, Iglesias A, Rojas MC (1992). Factores de riesgo en la transmisión de la hepatitis B en la Amazonía Colombiana.. Biomedica.

[39] Ramsey GH (1931). Fever with jaundice in the Province of Santa Marta, Colombia..

[40] Buitrago B, Hadler SC, Popper H, Thung SN, Gerber MA (1986). Epidemiologic aspects of Santa Marta hepatitis over a 40-year period.. Hepatology.

[41] Slusarczyk J (2000). Who needs vaccination against hepatitis viruses?. Vaccine.

[42] Ministerio de Salud (2002). Boletin Epidemiologico Semanal: Situacion de Hepatitis B en Colombia a la semana Epidemiologica.. http://www.col.ops-oms.org/sivigila/2002/BOLE49_02.pdf.

[43] Lai MM (1995). The molecular biology of hepatitis delta virus.. Annu Rev Biochem.

[44] World Health Organization (2001). Hepatitis Delta..

[45] Martinez M, De la Hoz F, Jaramillo LS, Rojas MC, Buitrago B (1991). Seroepidemiologia de la infeccion por el virus de la hepatitis B en ninos de la Amazonia Colombiana.. Biomedica.

[46] Buitrago B (1991). Historia natural de las hepatitis B y D en Colombia.. Biomedica.

[47] Schmunis GA, Zicker F, Pinheiro F, Brandling-Bennett D (1998). Risk for transfusion-transmitted infectious diseases in Central and South America.. Emerg Infect Dis.

[48] Ministerio de Salud (1996). Manual de normas técnicas, administrativas y de procedimientos..

[49] Schmunis GA, Cruz JR (2005). Safety of the blood supply in Latin America.. Clin Microbiol Rev.

